# Management of Ulnar Collateral Ligament Injuries of the Elbow

**DOI:** 10.1007/s12178-026-10042-x

**Published:** 2026-06-13

**Authors:** Patrick M. Ryan, Julia Beyer, Hayden Hartman, Humza Ismail, Mazin Alam, Steven F. DeFroda

**Affiliations:** Missouri Orthopaedic Institute, 1100 Virginia Ave, Columbia, MO 65203 USA

**Keywords:** Ulnar collateral ligament, UCL reconstruction, UCL repair, Internal brace, Overhead throwing athlete, Elbow instability

## Abstract

**Purpose of Review:**

Ulnar collateral ligament (UCL) injuries are increasingly prevalent among overhead throwing athletes, with reconstruction rates rising 193% from 2002 to 2011 and an annual 9% increase among adolescents. This review synthesizes current evidence on the anatomy, biomechanics, clinical evaluation, and management of UCL injuries, with a focus on comparative outcomes across nonsurgical and surgical treatment strategies.

**Key Findings:**

Nonsurgical management achieves return to sport in 80–100% of non-throwing athletes, while outcomes in overhead throwers are highly dependent on tear location, with proximal tears yielding superior success rates (89.7%) compared to distal tears (41.2%). Platelet-rich plasma remains controversial. UCL reconstruction achieves 80–97% return to play at 12–14 months; however, return to pre-injury performance is less consistent (67–87%). Primary repair with internal brace augmentation offers comparable return-to-play rates (98%) with a faster recovery timeline (9.2 versus 13.4 months) in appropriately selected patients with acute avulsion injuries.

**Summary:**

Treatment decisions should be individualized based on injury characteristics, tear location, tissue quality, and patient competitive goals. The proposed treatment algorithm stratifies management by tear location to facilitate evidence-based decision-making and realistic preoperative counseling.

## Anatomy

The ulnar collateral ligament is located at the medial aspect of the elbow and consists of 3 bundles: anterior, posterior, and transverse (inferior) [1–3]. The most prominent part is the anterior bundle, which is also the most well-defined bundle of the UCL. The anterior bundle originates from the anterior aspect of the medial epicondyle and inserts at the proximal coronoid process of the ulna. The posterior bundle is a fan-shaped bundle that is most prominently visible when the elbow is flexed to 90 degrees. It originates from the posterior aspect of the medial epicondyle and inserts on the medial aspect of the olecranon process. The posterior bundle serves as the floor of the cubital tunnel. The transverse bundle runs horizontally between the coronoid and the tip of the olecranon process and serves to connect the anterior and posterior bundles [3, 4]. 

## Biomechanics of the UCL

The elbow is a hinge joint that is restrained allows for flexion and extension while being restrained from varus and valgus rotation. The UCL is the primary restraint against valgus torque to the medial elbow [4]. The UCL also acts as a restraint against posteromedial rotary instability in the elbow joint [5]. Overhead throwing motions, common in baseball and tennis, have shown that the greatest force seen on the UCL occurs in the late cocking and early acceleration phase of the overhead motion [6]. Studies as early as 1983 reported the anterior portion of the UCL to be the primary restraint of the elbow against valgus stress from 30° to 120° of elbow flexion [3]. A more recent study in 2017 has shown that the UCL provides approximately one-third of valgus stability in full extension versus one-half of valgus stability at 90° of flexion [7]. The UCL also plays a large role in elbow stability in the setting of pronation and supination. A cadaver study in 2000 showed that when quantifying elbow instability with respect to internal and external rotation, UCL resection led to significantly laxity in passive flexion during supination [8]. As mentioned above, many studies have shown that the anterior portion of the UCL (AUCL) is the primary stabilizing portion of the UCL [4, 9]. The transverse portion of the UCL (transverse band) has traditionally been thought to have little to no effect on the function of the UCL. A 2011 study challenged these findings by reporting that the transverse band was not poorly developed as previously reported [5]. A 2021 cadaver study found that the transverse band played a significant role in the neutral zone of the elbow when resected, highlighting the importance of all three portions of the UCL in joint biomechanics [7]. The findings of previous biomechanical studies of UCL function highlight its importance in elbow stability and provide a pretext to explore best practices in the conservative and surgical management of UCL injuries to best restore its many roles in elbow stability and function.

## Injury Epidemiology

UCL injury is most prevalent amongst overhead throwing athletes, particularly baseball pitchers. The incidence has increased substantially, especially among adolescents with an 11-fold increase in high school pitchers [1, 10–13]. Risk factors include high pitch counts, multiple team participation, pitching while fatigued, glenohumeral internal rotation deficit, and loss of total arc of motion [14–17].

## Clinical Evaluation

### History

Clinical assessment of an athlete presenting with medial-sided elbow pain should begin with a detailed history, with particular attention to symptom onset, duration, and the phase of throwing motion during which the pain is experienced. Overhead throwers commonly report vague medial discomfort with declining velocity and accuracy. Associated ulnar nerve complaints, including paresthesia involving the small and ring finger, should be evaluated.

### Physical Exam

Physical examination includes visual inspection, palpation of the medial epicondyle and surrounding structures, and range of motion assessment. Focal tenderness at the sublime tubercle or medial epicondyle may indicate UCL injury. Throwing athletes commonly demonstrate mild flexion contractures of the dominant elbow. The ulnar nerve should be examined for instability or irritability throughout elbow motion.

To differentiate alternative causes of medial elbow pain -- including valgus extension overload, medial epicondylitis, and flexor-pronator mass injury -- provocative maneuvers such as resisted forearm pronation and forced elbow extension are performed [18]. Valgus stability should be assessed bilaterally. The static valgus stress test is typically performed with the elbow flexed to approximately 30°, preferentially loading the anterior bundle of the UCL. Examiner attention should be paid to pain reproduction or apprehension during these maneuvers, as small degrees of medial joint opening may be difficult to detect on physical examination alone.

## Imaging

### Radiographs

Standard anteroposterior, lateral, and oblique elbow radiographs should be obtained and carefully reviewed, as they may reveal subtle findings despite frequently appearing normal. Pertinent findings may include osseous abnormalities, UCL ossification, and osteophytes.

### Magnetic Resonance Imaging (MRI)

**Fig. 1:**
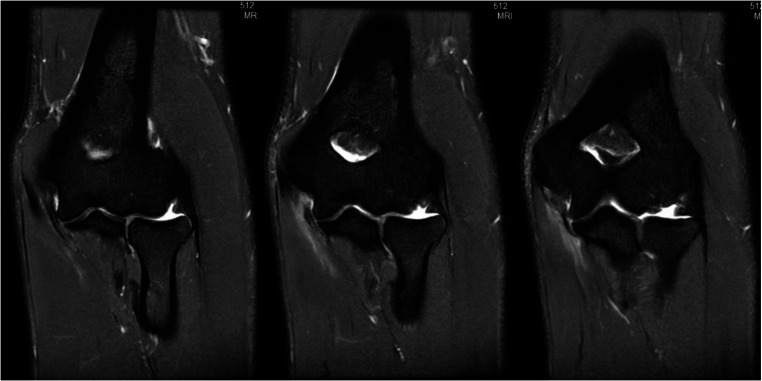
Coronal proton density weighted MRI series demonstrating a partial proximal tear of the ulnar collateral ligament

**Fig. 2:**
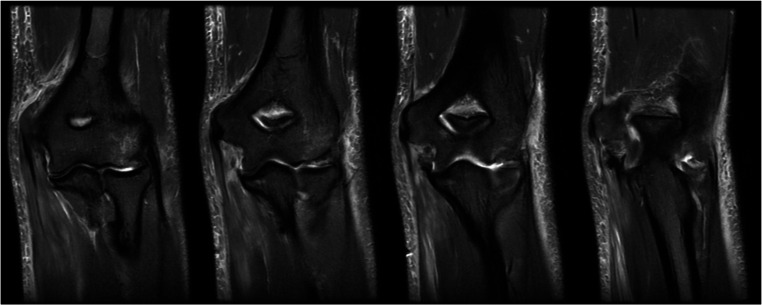
Coronal proton density weighted MRI series demonstrating a mid-substance tear of the ulnar collateral ligament

**Fig. 3:**
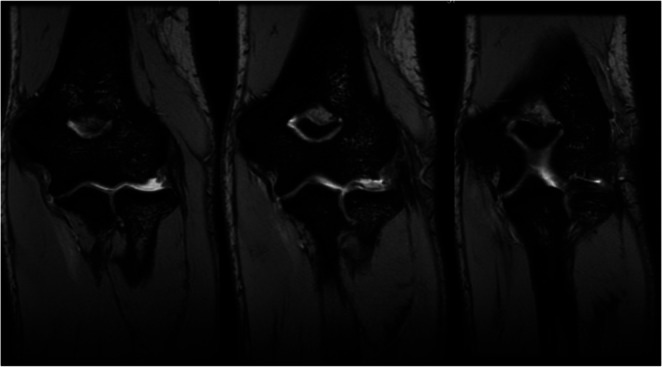
Coronal T2 MRI series demonstrating a distal tear of the ulnar collateral ligament

Magnetic resonance imaging (MRI), including magnetic resonance arthrography (MRA), represents the most reliable modality for detecting both partial- and full-thickness UCL disruptions, as well as associated intra-articular and periarticular pathology. MRI may demonstrate ligament thickening, increased signal, and discontinuity. MRA has demonstrated superior diagnostic accuracy compared with conventional MRI for identifying UCL injury (92% sensitivity, compared with 57%) with contrast extravasation around the site of the ligament tear being pathognomonic for UCL injury [8, 19]. Tears may be identified within the proximal (Fig. 1), midstance (Fig. 2) or distal aspect (Fig. 3) of the UCL.

## Management

### Non-Surgical Management

#### Indications

Indications include UCL injuries in non-throwing athletes, low functional demands, and partial thickness tears. An initial nonoperative trial of 3 months is recommended for most patients [2, 10, 20–25]. 

#### Rehabilitation

Conservative management begin with a 6–8 week period of rest, with ice and bracing. Progressive range of motion and strengthening exercises are then initiated, with gradual return to throwing with attention to mechanics and kinetic chain deficits [2, 21–23, 26].

#### Role for Biologics

Biologics have been explored as a component of management of UCL injuries, primarily in the form of platelet-rich plasma (PRP). PRP is typically utilized as a nonoperative adjunct [2, 10, 24]. Promising results have been reported for patients with partial tears [10, 21–24], however significant variability in PRP preparation and injection protocols necessitates that further high-quality data is necessary to elucidate the role of PRP in management of UCL injuries [2, 10, 22–24]. 

### Surgical Management

#### Reconstruction

**Fig. 4:**
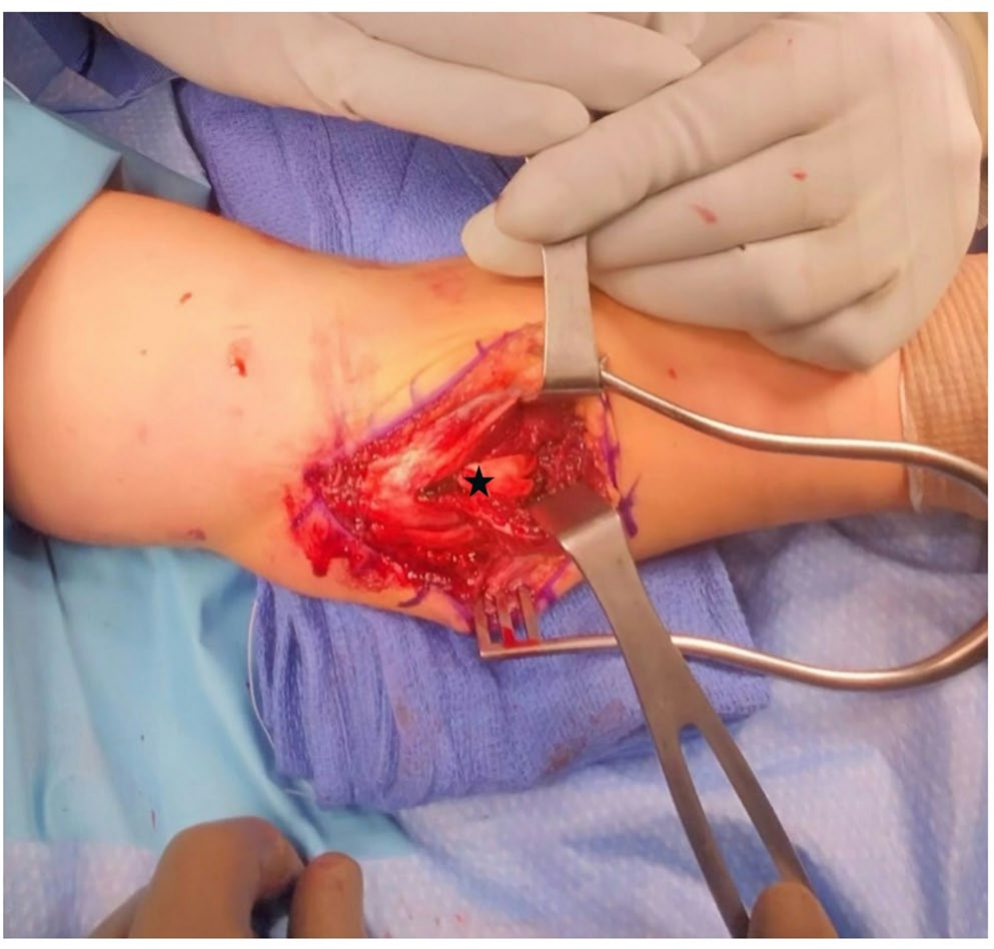
Intra-operative photograph of the medial aspect of a left elbow demonstrating a UCL reconstruction. The two limbs of the graft are identified by the black star

UCL reconstruction is widely considered the gold standard of surgical management [20, 27]. Indications include failed conservative management (typically 3 months), complete tears, and distal avulsions. Common techniques include the Jobe technique (figure of eight graft configuration through two humeral sided tunnels with ulnar nerve transposition), the modified Jobe technique (muscle splitting surgical approach), and docking (epicondyle-preserving via single humeral sided tunnel). Autografts include palmaris longus (most common), gracilis, or semitendinosus [2, 10, 20–22, 24, 28–31]. An operative image of a UCL reconstruction is demonstrated in Fig. 4.

#### Repair

**Fig. 5:**
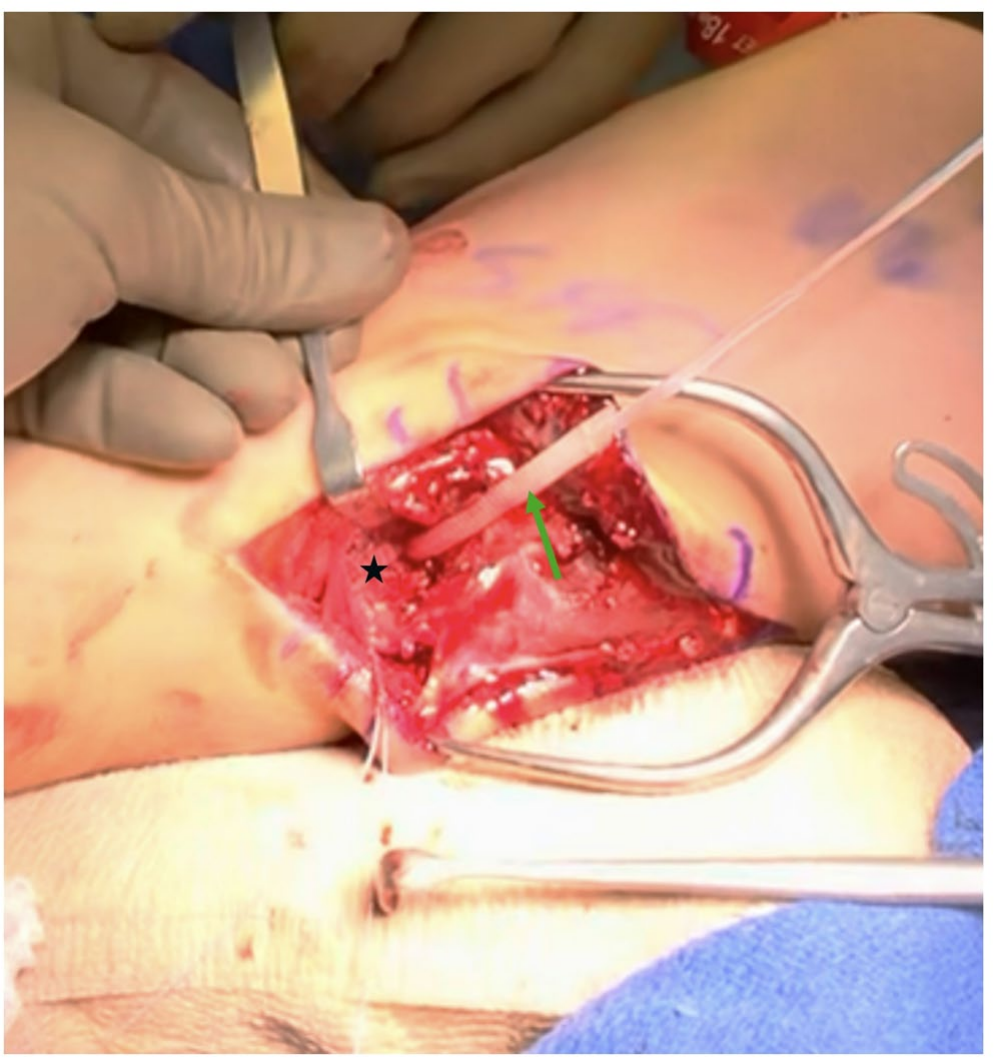
Intra-operative photograph of the medial aspect of a right elbow demonstrating a UCL repair with suture augmentation. The augmented fixation is demonstrated by the black star with excess tape following humeral sided fixation augmentation identified by the green arrow

UCL repair with augmentation offers shorter rehabilitation and faster return to sport [2, 20, 25, 32]. Augmented repair may be considered in acute proximal or distal avulsions in patients with good quality tissue [2, 10, 20, 32]. The typical candidate for UCL repair is a healthy young athlete who has failed a trial of nonoperative management [2, 25]. Repair may be considered for partial tears as well [2, 10, 25]. UCL repair is typically augmented with a collagen-coated suture tape, which has been shown to provide comparable biomechanical construct when compared to tendon to reconstruction [10, 20, 25, 32]. Following UCL repair, patients may follow an accelerated rehabilitation protocol [25]. An operative image of a UCL repair with suture tape augmentation is demonstrated in Fig. 5.

#### Hybrid Reconstructions

Hybrid reconstructions involve augmentation of ligament grafts with an internal brace in order to improve biomechanical strength and attempt to shorten return to play time [20, 33]. Augmented reconstructions have demonstrated increased load to failure in comparison to non-augmented reconstructions in biomechanical studies [20]. 

### Rehabilitation and Return to Sport

Optimizing outcomes after ulnar collateral ligament (UCL) injury requires structured, phase-specific rehabilitation that balances biological tissue healing with the progressive restoration of kinetic chain mechanics [25]. Transition through rehabilitation phases is strictly criterion-based, rather than timeline-dependent, to minimize the risk of attenuated or catastrophic construct failure [25]. 

### Non-Operative Rehabilitation and RTS

Non-operative management focuses on resolving acute inflammation, protecting the healing ligament from valgus stress, and addressing underlying biomechanical deficits such as glenohumeral internal rotation deficit (GIRD) [14]. Phase I (Acute/Protection) lasts 2–6 weeks depending on severity; the elbow is protected in a hinged brace to avoid terminal extension valgus stress while hand, wrist, and shoulder gripping/strengthening are initiated immediately [21]. Phase II (Subacute/Progressive Motion) focuses on achieving full passive and active range of motion (ROM) and strengthening the flexor-pronator mass, which serves as the dynamic stabilizer against valgus stress [9, 21]. Phase III (Functional/Return to Throwing) begins upon completion of a structured non-operative trial (typically 6–8 weeks of pain-free activity) [21, 25]. Prior to starting a multi-stage interval throwing program (ITP), the athlete must successfully pass a formal functional assessment demonstrating symmetric, pain-free terminal extension, equivalent bilateral upper extremity strength, and absolute resolution of medial tissue tenderness. [21] Unrestricted competitive return to sport requires completion of the ITP without symptom recurrence and restoration of full pre-injury throwing velocity and accuracy [21, 34]. 

### UCL Reconstruction (UCLR) Rehabilitation and RTS

Traditional UCLR rehabilitation undergoes a deliberate, prolonged protocol to accommodate the slow revascularization and ligamentization of the free tendon autograft [22, 27]. Phase I (Weeks 0–4) protects the elbow in a hinged brace locked at 90° for the first week, followed by progressive ROM allowance to 120° by week 4, while avoiding shoulder external rotation to limit distraction valgus forces [27]. Phase II (Weeks 5–12) normalizes terminal extension and flexion, transitioning out of the brace by week 6 to aggressively advance isotonic strengthening of the forearm flexors, pronators, and shoulder stabilizers [22, 27]. Phase III (Months 4–9) focuses on advanced sports-specific plyometrics and eccentric upper extremity training [22]. An interval throwing program typically begins no earlier than 6 months postoperatively [11]. Criteria for eventual competitive clearance include pain-free clinical examination with absolute symmetry in passive elbow ROM, documented rotator cuff and periscapular strength testing within 90–100% of the contralateral limb, and successful, pain-free progression through a comprehensive ITP spanning bullpen sessions and live simulated games [11, 27, 28]. Full return to unrestricted competition averages 12 to 14 months [11, 27]. 

### UCL Repair with Internal Brace Augmentation Rehabilitation and RTS

The intrinsic biomechanical stability provided by collagen-coated suture tape augmentation permits an accelerated rehabilitation timeline by safeguarding the native tissue repair against early failure [20, 35, 36]. Phase I (Weeks 0–2) emphasizes immediate postoperative mobilization, placing the elbow in a hinged brace allowing an early range of motion from 10° to 120° with immediate submaximal isometric forearm and shoulder exercises [25, 35]. Phase II (Weeks 3–8) discontinues the hinged brace by week 3 to 4 to introduce full dynamic upper extremity strengthening, advanced flexor-pronator mass loading, and rotational kinetic chain integration [25, 37]. Phase III (Weeks 9–12) completes aggressive plyometric training and dynamic shoulder stabilization [25]. Because native proprioception and structural anatomy are preserved, athletes undergoing augmented repair can safely transition to an ITP as early as 10 to 12 weeks postoperatively [4, 37]. Competitive clearance is granted once the patient meets the identical objective strength, motion, and functional benchmarks required for reconstruction [20, 37]. The mean timeline for unrestricted competitive return to sport after augmented repair is significantly abbreviated, typically ranging from 5 to 7 months [26]. 

## Outcomes

### Nonsurgical Management

In non-throwing populations—such as wrestlers, gymnasts, and football players—outcomes following nonsurgical management are generally excellent due to the acute, traumatic nature of injury rather than the chronic attrition, as is generally seen in pitchers. A recent systematic review and multicenter study of non-throwing athletes demonstrated successful return to sport rates of approximately 80% to 100% with conservative care alone [34]. 

#### Overhead Throwing Athletes

In overhead throwing athletes, nonsurgical outcomes are highly dependent on the location and tear grade. While conservative management was historically viewed with skepticism for high-level throwers, modern protocols utilizing platelet-rich plasma (PRP) have shown variable results. A systematic review found an overall return to sport (RTS) rate of 79.7% for athletes treated nonoperatively, with patient possessing proximal UCL tears achieving a significantly higher return to sport rate (89.7%) compared to those with distal tears (41.2%) [38]. For professional baseball pitchers with Grade 1 injuries, RTS rates can approach 100%, declining to 66–94% for Grade 2 injuries [11]. 

The role of PRP in UCL management remains controversial. Recent studies show conflicting results, with some demonstrating potential delays in return to sport [39]. Current evidence suggests PRP may be considered in select patients with partial-thickness tears [40, 41]. 

### Ulnar Collateral Ligament Reconstruction

#### Return to Play Rates

Following UCL reconstruction, outcomes in professional baseball pitchers are well-documented. After primary UCLR, 80–97% of Major League Baseball (MLB) pitchers successfully returned to play in approximately 12 months; however, return to prior level of play was between 67 and 87% at approximately 15 months [27]. Moreover, recent data has reported most MLB pitchers return at 2 years (79%) rather than 1 year (4%), with mean return time of 558 days [42]. 

#### Performance Metrics

While RTS rates are generally favorable, return to the same level of performance is less certain. Approximately 80% of MLB pitchers return to the major league level, however many experience reduced workload and velocity during their first post-operative season [11]. A systematic review found that while MLB players returned to play 89% of the time and returned to the same level of play 78% of the time, worse in-game pitching statistics, decreased innings pitched, and decreased fastball velocity after UCL injuries were appreciated [27]. Recent investigations utilizing advanced performance metrics have provided more granular insights into post-operative performance. Studies show considerable decreases in innings pitched and workload in postoperative years 1–2, though per-inning effectiveness may be preserved [42]. Meanwhile, MLB pitchers demonstrate decreased wins above replacement (WAR) and runs above replacement (RAR) compared to pre-UCLR levels, though fastball velocity is typically similar upon return [42, 43]. 

#### Timing of Surgery and Career Impact

The timing of UCL-R on career length in pitchers appears to have notable implications for both recovery and long-term outcomes. A recent study of elite pitchers selected in the first 5 rounds of the MLB Draft from 2011 to 2020 found that of the 851 pitchers selected, 281 (33.0%) had undergone primary UCL-R. Compared to early-career UCL-R pitchers, median MLB career earnings (adjusted for inflation) were significantly higher for middle-career and late-career UCL-R pitchers even after adjustment for confounders, including age. Furthermore, UCL-R return to sport recovery time (any level) for primary reconstruction was significantly longer for early-career UCL-R compared to late-career UCL-R pitchers (19.79 ± 5.69 vs. 15.36 ± 3.45 months; *p* = 0.023). These findings suggest that early-career UCL-R may be associated with lower MLB career earnings and prolonged recovery times, potentially due to the mental health burden and developmental disruption in younger athletes [23]. 

#### Collegiate Pitchers

Collegiate pitchers achieve 79–81% return to sport with no significant differences in postoperative statistics compared to matched controls [28]. 

#### Non-Throwing Athletes

Non-throwing athletes achieve 100% RTS at 10 months [34]. 

#### Surgical Technique

RTS rates are comparable between docking and modified Jobe techniques, though docking shows lower ulnar nerve complications (6.0% vs. 19–29%) [44]. 

#### Position Players

UCLR in position players has been reported to yield faster return to sport timing than pitchers (9.5 vs. 12–14 months), though catchers have lower success rates [11, 13]. Specifically, RTS rates for catchers ranges from 59% to 80%, compared to 76% for infielders and 89% for outfielders. This disparity is likely attributable to the high frequency and intensity of throwing required in the catching position, which may exceed the biomechanical capacity of the reconstructed ligament [27]. A study of 26 MLB position players who underwent UCL reconstruction found that 80% returned to their preinjury level of competition, with performance metrics including WAR, on-base plus slugging (OPS), and isolated power (ISO) showing no significant decreases compared to preoperative performance and matched controls. These findings suggest that position players can reasonably expect to return to their preinjury level of competition and performance after surgery, though catchers represent a higher-risk subgroup that warrants specific counseling [27]. 

### Primary UCL Repair with Internal Brace Augmentation

UCL repair with internal bracing offers accelerated recovery for proximal or distal avulsions by preserving native anatomy [35, 37]. 

#### Time to Return to Sport

The most significant advantage of UCL repair with internal brace is the shortened time to return to sport. In a 2025 study comparing UCL reconstruction versus repair, two groups differed significantly regarding time to return to practice (6.7 ± 3.5 months for repair vs. 10.2 ± 11.7 months for reconstruction, *p* < 0.01) and time to return to competition (9.2 ± 4.6 months for repair vs. 13.4 ± 13.3 months for reconstruction, *p* < 0.01) [4]. Earlier studies reported that athletes undergoing repair returned to competition at an average of 6.7 months, compared to 10.2–13.4 months for reconstruction [35]. In a prospective study of 66 overhead athletes, 96% (54/56) of those who desired to return to the same or higher level of competition were successfully at an average of 6.1 months (range 3.2–12 months), with 65% returning in less than 6 months [37]. 

Smaller investigations have corroborated these findings. One study reported a mean return time of 9.2 months for repair patients [45], while a systematic review found that the average time required to return to sport after UCL repair with internal brace to be approximately 5–7 months [33]. These data collectively demonstrate that UCL repair with internal brace offers a clinically significant advantage in terms of return-to-play timeline, which may be particularly valuable for athletes with limited windows of opportunity or competitive eligibility.

#### Non-Throwing Athletes

In non-throwing athletes, UCL repair with internal brace has similarly demonstrated high efficacy. A study of 40 non-throwing athletes demonstrated a 93% RTS rate with the average time to play occurring at 7.4 months [34]. 

**Fig. 6:**
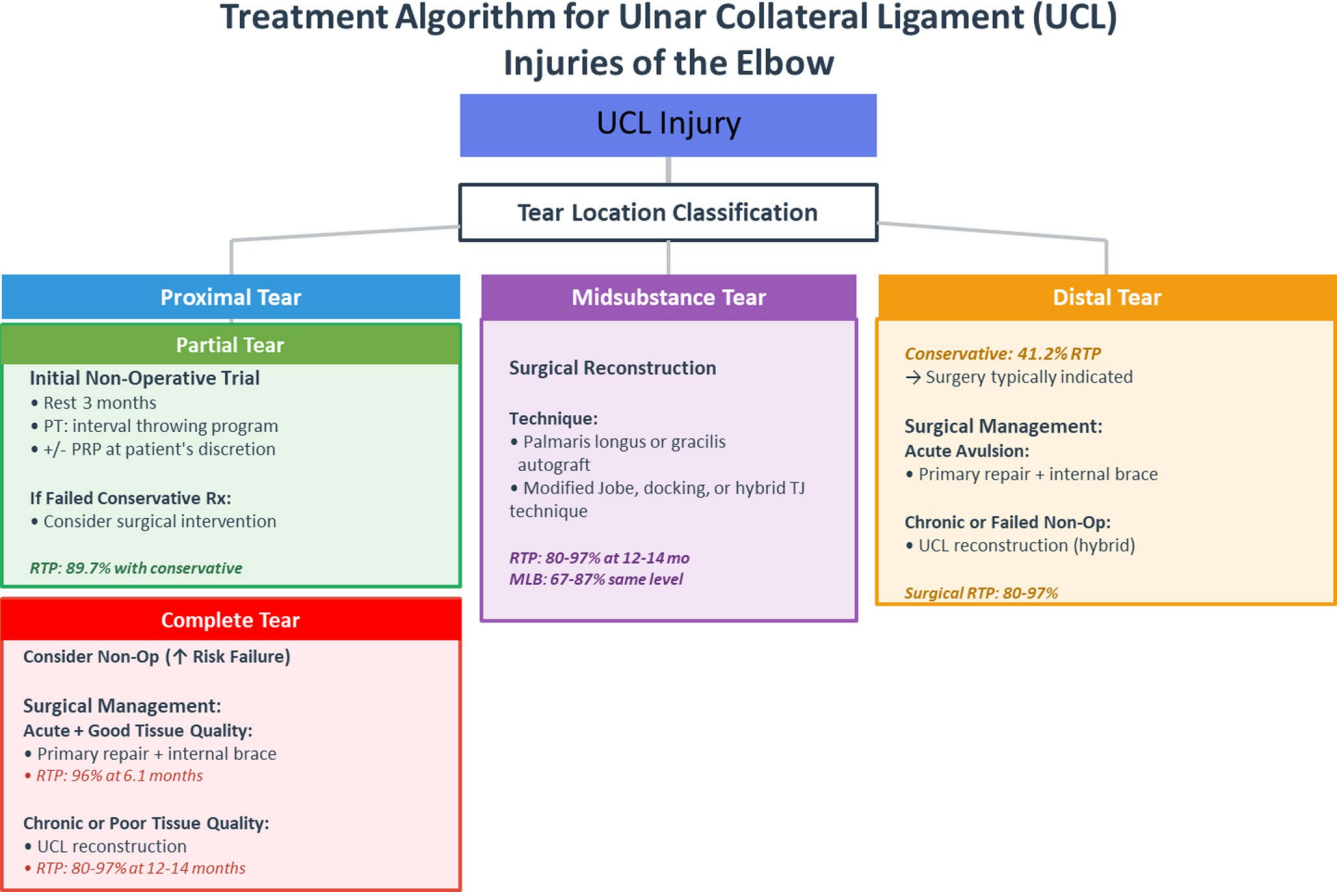
Treatment algorithm for UCL injuries stratified by tear location with surgical decision-making based on tear characteristics, tissue quality, and chronicity

Based on the aforementioned data, we propose a treatment algorithm for management of UCL injuries based on location of the tear (Fig. 6). For proximal injuries, an initial nonoperative trial is recommended in the setting of partial tears (Fig. 2). For complete proximal tears, nonoperative management may have an increased risk of failure. Therefore, primary repair and internal brace is recommended in patients with acute injuries and good tissue quality. For patients with chronic injuries or poor tissue quality, UCL reconstruction is recommended. For mid-substance tears (Fig. 3), surgical reconstruction is recommended. For distal tears (Fig. 4), surgical management is recommended with primary repair and internal brace for acute avulsions or UCL hybrid reconstruction for chronic injuries or those who failed initial nonoperative management.

## Conclusion

UCL injury outcomes depend on treatment modality and patient selection. Nonsurgical management succeeds in select patients with proximal tears. Primary reconstruction achieves 80–97% RTS though workload may decrease. Repair offers faster recovery (9 vs. 13 months) for avulsion injuries. Revision procedures yield inferior outcomes (55–78% return to previous level) [11, 13, 26–28, 35, 37, 38, 40–42, 44, 46].

## Key References

- Spears et al., JAAOS 2025
  - ◌ Excellent comprehensive surgical UCL review, synthesizing reconstruction, repair, and hybrid augmentation under one framework. Key reference because it directly grounds the treatment algorithm in this manuscript and represents the highest-level contemporary evidence spanning all technique categories. 
- Gehrman et al., Am J Sports Med 2024
  - ◌ The most current large-scale epidemiologic analysis of UCL reconstruction practice patterns in MLB pitchers, including real-world shifts in graft selection and augmentation use. Key reference because it contextualizes the evolving surgical landscape described in the review and provides a dataset on technique-level trends at the professional level.
- Mastroianni et al., Am J Sports Med 2025
  - ◌ Current return-to-performance analysis in MLB pitchers post-UCL surgery, using modern pitch-tracking metrics (Stuff+, Location+, Pitching+, fWAR) unavailable in earlier studies. Key reference because its finding that only 28% of pitchers fully recover pre-injury performance at 3 years is the strongest recent evidence supporting the cautious counseling message in this review's outcomes and summary sections.

## Data Availability

No datasets were generated or analysed during the current study.
